# AI Applications in Depression Detection and Diagnosis: Bibliometric and Visual Analysis of Trends and Future Directions

**DOI:** 10.2196/79293

**Published:** 2025-10-22

**Authors:** Wenbo Ren, Xiali Xue, Lu Liu, Jiahuan Huang

**Affiliations:** 1 Center for Rehabilitation Medicine, Rehabilitation & Sports Medicine Research Institute of Zhejiang Province Department of Rehabilitation Medicine Zhejiang Provincial People’s Hospital (Affiliated People’s Hospital), Hangzhou Medical College Hangzhou, Zhejiang China; 2 Department of Rehabilitation Medicine Peking University Third Hospital Beijing China; 3 Department of Cardiology Second Affiliated Hospital, Zhejiang University School of Medicine Hangzhou China; 4 Geriatric Medicine Center Department of Geriatric Medicine Zhejiang Provincial People’s Hospital, Affiliated People’s Hospital, Hangzhou Medical College Hangzhou, Zhejiang China

**Keywords:** artificial intelligence, bibliometrics, computational psychiatry, depression diagnosis, digital health, machine learning, mental health, psychiatry

## Abstract

**Background:**

Depression is a highly prevalent and debilitating mental disorder, but its diagnosis largely relies on subjective assessments, creating challenges in accuracy and consistency. Advances in artificial intelligence (AI) offer promising avenues for more objective and efficient diagnostic approaches. Understanding the evolving landscape of AI applications in depression diagnosis is essential for guiding future research and clinical translation.

**Objective:**

This study aims to provide a comprehensive bibliometric and visual analysis of the global research trends, intellectual structure, and emerging frontiers in the application of AI for depression detection and diagnosis from 2015 to 2024.

**Methods:**

We conducted a systematic literature search in the Web of Science Core Collection database to identify publications on AI applications in depression diagnosis from January 1, 2015, to December 31, 2024. A total of 2304 articles were retrieved and analyzed using bibliometric software CiteSpace. The analysis encompassed temporal trends, keyword dynamics, author collaboration networks, institutional influence, country contributions, and intellectual foundations through co-citation analysis of journals and references.

**Results:**

The field exhibited exponential growth in publications and citations, particularly after 2018, reflecting increasing academic and clinical interest. Key thematic shifts were observed from traditional machine learning to advanced deep learning, multimodal fusion, and the integration of objective biomarkers (eg, electroencephalography, facial expressions). Leading contributors included institutions from China and the United States, with collaborative links also forming with countries such as Canada and Singapore. The intellectual base is highly interdisciplinary, drawing heavily from computer science, neuroscience, and psychiatry, with a notable surge in engineering and translational research.

**Conclusions:**

The integration of AI in depression diagnosis is a rapidly maturing and diversifying field, transitioning from theoretical exploration to clinically relevant applications that emphasize objective, data-driven approaches. The identified trends underscore the need for enhanced interdisciplinary and international collaboration, the development of ethical frameworks, and a focus on translating technological innovations into accessible and equitable mental health solutions. These findings offer valuable insights for researchers, clinicians, and policy makers to strategically advance AI-assisted depression diagnostics globally.

## Introduction

Depression is a highly prevalent and debilitating mental disorder, characterized by a prolonged disease course, high recurrence rates, and a significant risk of suicidal ideation in severe cases [1,2]. According to the World Health Organization (WHO), depression affects an estimated 350 million people worldwide and is projected to become the leading cause of disability by 2030 [3,4]. This alarming prevalence underscores the urgent need for more effective diagnostic tools.

Current clinical diagnosis largely depends on subjective, self-reported measures and rating scales. The use of these tools is often limited by inherent subjectivity and issues with patient adherence [5,6]. Furthermore, the pronounced heterogeneity of depressive symptoms and the pervasive issue of patient underreporting render the timely and precise detection of depression a formidable clinical challenge. This exigency unequivocally underscores the urgent need for more objective, data-driven diagnostic approaches [7-9].

In recent years, the integration of artificial intelligence (AI) has emerged as a promising frontier for advancing depression detection and diagnosis [10]. Technologies such as machine learning, deep learning, natural language processing, and computer vision have been increasingly used to analyze diverse multimodal data, including speech patterns, facial expressions, and neuroimaging data [11-13]. However, the existing research remains somewhat fragmented. Key challenges persist, including inconsistencies in evaluation metrics and a notable lack of generalizability across diverse populations and technological platforms.

A robust understanding of the field’s developmental trajectory—encompassing key contributors, leading collaborative institutions, and topics gaining substantial momentum—is therefore essential for strategically guiding future research endeavors and informing effective policy directions. To address this critical knowledge gap, this study undertakes a systematic bibliometric and visual analysis of the global research landscape on AI applications in depression diagnosis from 2015 to 2024. Among available bibliometric tools, CiteSpace offers unique methodological strengths, including the ability to detect emerging trends, research frontiers, and intellectual turning points through co-citation analysis, burst detection, and betweenness centrality [14]. This level of analytical granularity is particularly well-suited to the domain of AI-driven depression research, where technological advances and interdisciplinary collaboration evolve rapidly. Unlike traditional narrative or systematic reviews, this study uses a bibliometric and visualization approach to systematically map the intellectual structure, collaborative networks, knowledge base, and emerging frontiers of AI applications in depression detection and diagnosis. Rather than reinterpreting individual findings, our analysis integrates the global body of literature to reveal hidden dynamic trends and potential interconnections across the field. The innovation of this study therefore lies in providing a panoramic perspective that not only confirms the rapid growth of the domain but also uncovers structural patterns and emerging directions not accessible through conventional review methodologies. These insights provide a valuable reference for interdisciplinary researchers, clinicians, and policy makers, thereby guiding future research and clinical translation.

## Methods

### Data Source and Retrieval Strategy

The bibliometric dataset for this study was systematically extracted from the Web of Science Core Collection, recognized as an authoritative global database for academic literature. To ensure the inclusion of high-quality, peer-reviewed research, the search was restricted to the Science Citation Index Expanded.

A comprehensive topic search string was developed and implemented through the advanced search interface. This query was designed to precisely capture publications relevant to AI applications in the detection and diagnosis of depression. The exact search query used was: TS = ((“depression” OR “major depressive disorder” OR “MDD”) AND (“artificial intelligence” OR “machine learning” OR “deep learning” OR “natural language processing” OR “NLP” OR “support vector machine” OR “neural network” OR “random forest” OR “decision tree” OR “convolutional neural network” OR “CNN”) AND (“diagnosis” OR “detection” OR “identification” OR “screening”)) AND LA=(English).

### Search String Validation and Sensitivity Analysis

To ensure the comprehensiveness and rigor of our literature search, the search string was developed through an iterative process. We initially constructed a set of keywords based on a preliminary review of the field’s seminal papers. This initial string was then refined by a domain expert to include a broader range of synonyms and related terms.

Furthermore, we conducted a sensitivity analysis to validate the robustness of our search strategy. Two alternative search strings with slightly different combinations of keywords were tested. A comparison of the retrieval results showed that while the total number of papers varied slightly, the core set of highly cited articles and the overall temporal trends remained consistent. This analysis confirms that our primary search string is robust and effectively captures representative publications in the field.

### Literature Search and Selection

We conducted a comprehensive literature search in the WoSCC, focusing on publications from 2014 to 2024 related to AI-assisted depression diagnosis in adolescents. The search string was iteratively developed and refined by domain experts to include relevant synonyms and related terms. To ensure robustness, we performed a sensitivity analysis using alternative search strings.

Duplicates were automatically removed and verified manually. Titles and abstracts were independently screened by 2 reviewers, and any disagreements were resolved through discussion or adjudication by a third reviewer. Full texts were then assessed for eligibility according to prespecified inclusion and exclusion criteria. The final dataset included 2304 publications.

**Figure 1:**
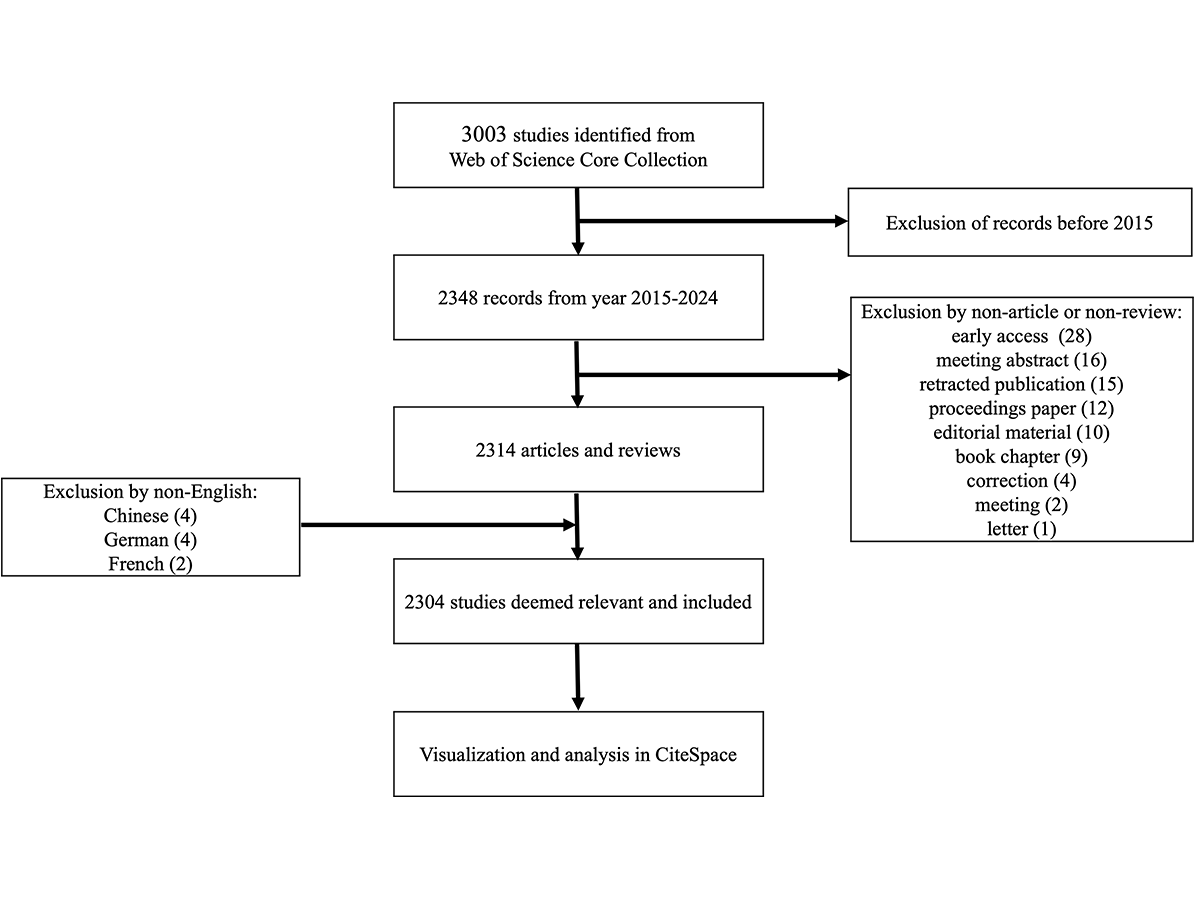
Literature screening flow diagram. Note: The exclusion categories are not mutually exclusive.

To enhance clarity and transparency, we added a flow diagram (Figure 1) to visually summarize the screening and selection process, showing the number of records identified, duplicates removed, records screened, full texts assessed, and studies included.

The bibliometric visualization and analysis in this study were conducted using CiteSpace (version 6.3.R1, 64-bit Basic), a widely recognized tool for detecting research trends, intellectual structures, and emerging topics through co-occurrence and co-citation networks [15]. To ensure analytical consistency and replicability, a unified parameter configuration was adopted across all modules.

The time span was set from 2015 to 2024, segmented into 1-year slices to allow temporal tracking of research dynamics. The term source was set to Title, Abstract, Author Keywords, and Keywords Plus, and “Pathfinder” and “Pruning sliced networks” were applied to remove redundant links and optimize visual clarity.

### Bibliometric Analytical Modules

Multiple types of nodes were analyzed, each corresponding to a specific dimension of the research landscape. Keywords were used to extract thematic clusters, hotspot evolution, and burst terms; cited authors, cited references, and cited journals were analyzed to identify intellectual foundations and knowledge flow; and authors, institutions, and countries were used to map scientific collaboration patterns.

The primary metrics for network evaluation included betweenness centrality, which detects pivotal nodes that bridge disparate clusters; citation burst, which highlights nodes with sudden surges in influence; and modularity (Q) and silhouette score (S), which evaluate cluster validity and cohesion.

All generated visualizations were exported in high resolution and manually optimized (eg, node labeling, color coding by year) to ensure clarity, interpretability, and consistency with publication standards. The combined use of structural and temporal metrics allowed for both snapshot-level analysis and longitudinal mapping, offering a comprehensive view of the field’s evolution.

### Network Evaluation Metrics

Structural metrics include frequency (F), which is the raw count of occurrences or citations and indicates research volume and impact magnitude; betweenness centrality, which quantifies a node’s role as an intermediary in network information flow, with values >0.1 indicating structural importance as knowledge brokers; and closeness centrality, which measures the average shortest path distance from a node to all other nodes, reflecting information accessibility.

Clustering quality metrics include Q, which assesses the strength of network division into distinct communities, with values >0.3 indicating significant cluster structure and higher values suggesting better-defined research communities; S, which evaluates cluster homogeneity and separation, with values >0.5 indicating high intracluster similarity and intercluster distinctiveness, and S >0.7 representing excellent clustering quality; and mean silhouette value, which is the average silhouette score across all clusters and provides an overall assessment of clustering quality.

### Temporal Analysis Metrics

Citation burst strength is calculated using Kleinberg’s algorithm, which detects abrupt increases in citation frequency, with burst strength indicating the magnitude of attention surge. Burst duration refers to the time period during which elevated attention persists, revealing the temporal scope of research interest. Sigma (σ) score is a composite metric combining citation frequency with burst strength, identifying publications with both sustained impact and sudden attention.

All generated charts and tables were meticulously exported in high resolution for subsequent postprocessing and manual labeling, ensuring optimal clarity, visual consistency, and publication-ready quality throughout the manuscript.

### Ethical Considerations

This study involved a bibliometric analysis of publicly available, deidentified bibliographic data. As no human participants were involved, formal ethical approval and informed consent were not required. All data were accessed and analyzed in accordance with relevant data privacy guidelines for public academic databases.

## Results

### Temporal Trajectory Analysis of Academic Impact

**Figure 2:**
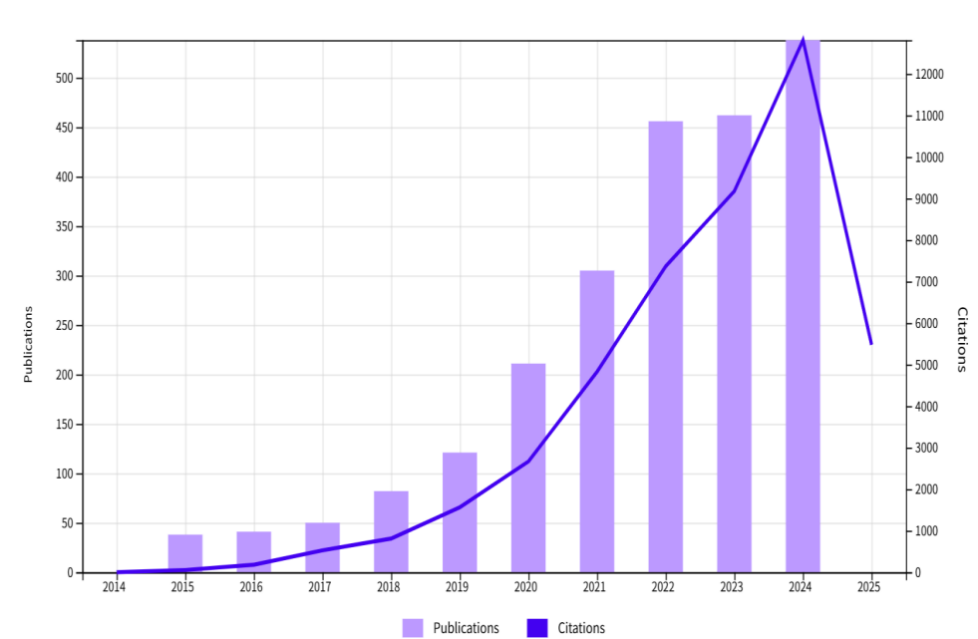
Longitudinal growth of artificial intelligence–based depression research: a decade of escalating publications and citations (2015-2024).

Table 1 and Figure 2 delineate the temporal evolution of scholarly output and citation impact related to the identification and diagnosis of AI-associated depression over the decadal period 2015-2024. The bar chart includes data from 2014 and 2025, which fall outside the predefined analysis window (2015-2024). These entries were automatically captured by the software due to early indexing or system defaults. As such, they were excluded from the core statistical interpretation but retained in the visualization for completeness. Data illustrate the annual publication volume alongside corresponding citation frequencies. These trends collectively provide a quantitative measure of the research community’s engagement and the escalating scientific interest in this domain. From 2015 to 2024, research output related to AI in depression studies experienced exponential growth. The number of annual publications rose from 38 in 2015 to 538 in 2024, marking a more than 13-fold increase. Similarly, citations surged dramatically—from 52 in 2015 to 12,812 in 2024—indicating a sharp rise in scholarly impact.

**Table 1:** Scholarly productivity and impact trajectories.

Years	Publications, n	Citations, n
2015	38	52
2016	41	180
2017	50	526
2018	82	811
2019	121	1564
2020	211	2668
2021	305	4829
2022	456	7368
2023	462	9175
2024	538	12,812

### Countries and Regions

**Figure 3:**
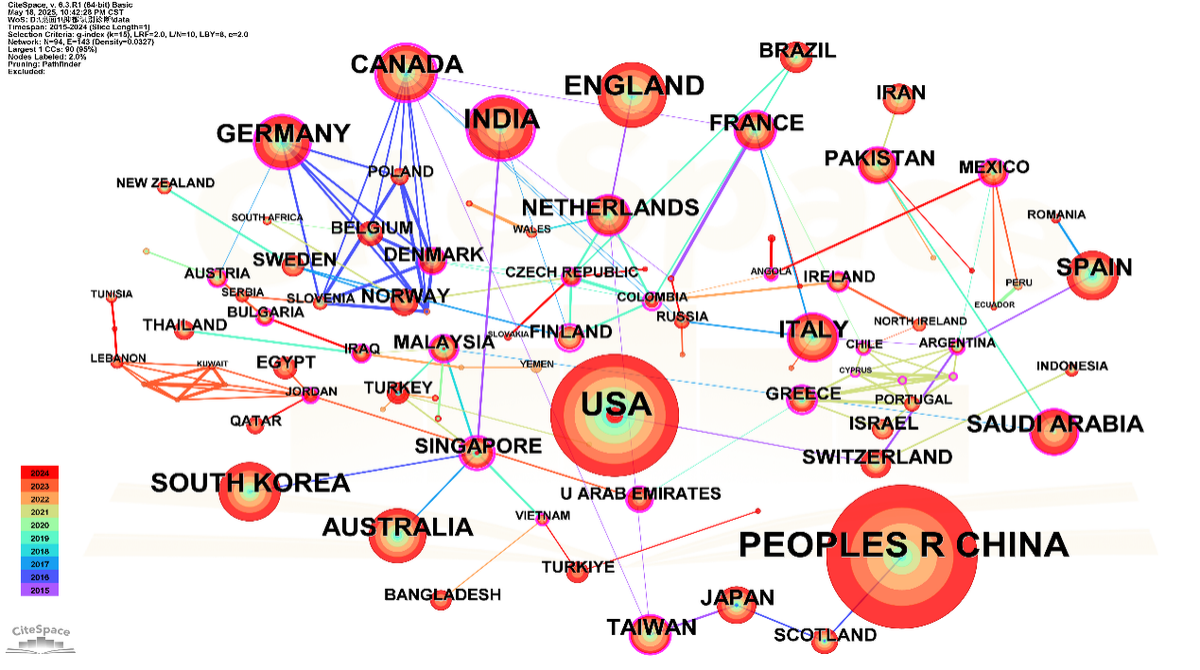
Country and region collaboration network.

The international collaboration network on AI applications in depression diagnosis from 2015 to 2024, as visualized in Figure 3 and detailed in Table 2, demonstrates both concentrated productivity and structural imbalance. China and the United States jointly accounted for over 62% (1331/2114) of total publications, firmly establishing their leadership in research output. However, both countries exhibited zero betweenness centrality, indicating a paradox between publication volume and network influence. In contrast, India (centrality=0.53), Canada (0.57), and Singapore (0.57) emerged as key knowledge intermediaries, occupying strategic bridging positions that link a substantial portion of the collaboration network, thereby facilitating knowledge exchange across countries. Temporal trends reveal a shift from early dominance by traditional research hubs such as Germany, Canada, and the United Kingdom (2015-2018) to increased participation by emerging economies, including Saudi Arabia and Pakistan, after 2019. This evolution reflects broader geographical diversification of scientific leadership and underscores the growing role of non-Western countries in advancing computational mental health research.

**Table 2:** Global collaboration metrics in artificial intelligence–driven depression research.

Country and region	Frequency, n	Centrality	Burst strength	Year span	Remarks
China	776	0.00	—^a^	2015-2023	Core producer, low brokerage
United States	555	0.00	8.92^b^	2015-2023	North American innovation hub
India	157	0.53	4.31	2015-2023	Highest structural holes
Canada	125	0.57	—	2015-2023	Transatlantic knowledge flow
Taiwan, China^c^	64	0.14	1.24	2017-2022	Specialized in EEG^d^ analytics
South Korea	139	0.39	5.12^e^	2017-2023	Digital phenotyping leader

^a^Not available.

^b^Calculated using Kleinberg’s algorithm (γ=0.8). Indicates significance (*P*<.05, Fisher exact test).

^c^Taiwan, China: Data from Taiwan is classified under “Taiwan, China” according to World Health Organization standards and United Nations Resolution 2758.

^d^EEG: electroencephalogram.

^e^Total collaboration frequency: 2114 (links with relative frequency [LRF]≥3, pruned isolated nodes).

### Institutions

**Figure 4:**
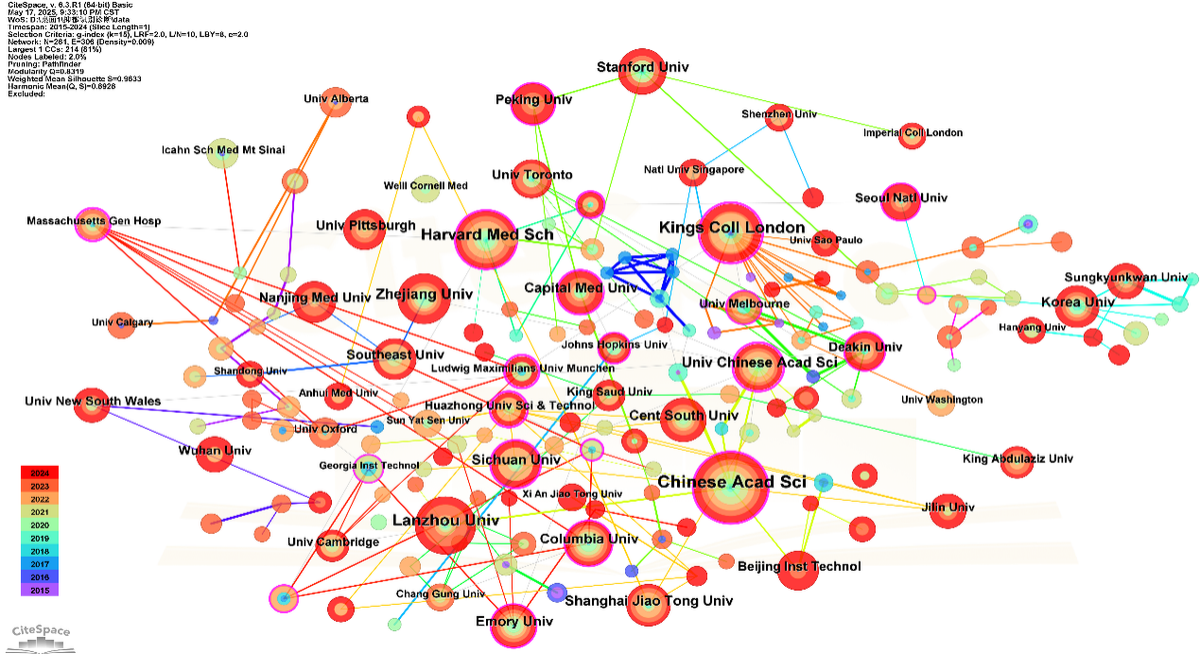
Institutional collaboration analysis.

The institutional collaboration network (Figure 4 and Table 3), based on 2304 publications from 2015 to 2024, reveals a well-structured and increasingly globalized research landscape. The visualization presents 214 institutions connected by 306 coauthorship links, with leading entities such as the Chinese Academy of Sciences, Harvard Medical School, and the University of Chinese Academy of Sciences emerging as dominant hubs in both productivity and network centrality. Notably, the Chinese Academy of Sciences exhibited the highest frequency (n=42) and the greatest centrality (0.14), underscoring its pivotal role in driving AI-assisted depression diagnostics. Harvard Medical School and Capital Medical University follow closely, demonstrating strong transnational collaborative ties. Regionally, the network shows multipolar connectivity, with active clusters in East Asia, North America, and Europe. Institutions such as Columbia University and the University of Toronto have gained increasing prominence in recent years, particularly after 2018, reflecting the growing momentum of AI-driven mental health research. European universities such as Oxford and Cambridge also play connecting roles despite moderate publication output. A significant shift toward more diversified, cross-border institutional partnerships is evident in the later stages of the decade, with institutions including the University of Toronto and the University of Melbourne becoming increasingly active after 2018.

**Table 3:** Summary table of core institutions.

Institution name	Count, n	Centrality	First year of appearance	Rank
Chinese Academy of Sciences	42	0.14	2015	1
Harvard Medical School	39	0.13	2016	2
University of Chinese Academy of Sciences	36	0.09	2017	3
Capital Medical University	32	0.11	2017	4
University of Toronto	29	0.10	2018	5
University of Melbourne	27	0.08	2018	6
University of Oxford	25	0.07	2016	7
Columbia University	24	0.06	2019	8
Zhejiang University	23	0.08	2020	9
Stanford University	22	0.06	2016	10

### Author

**Figure 5:**
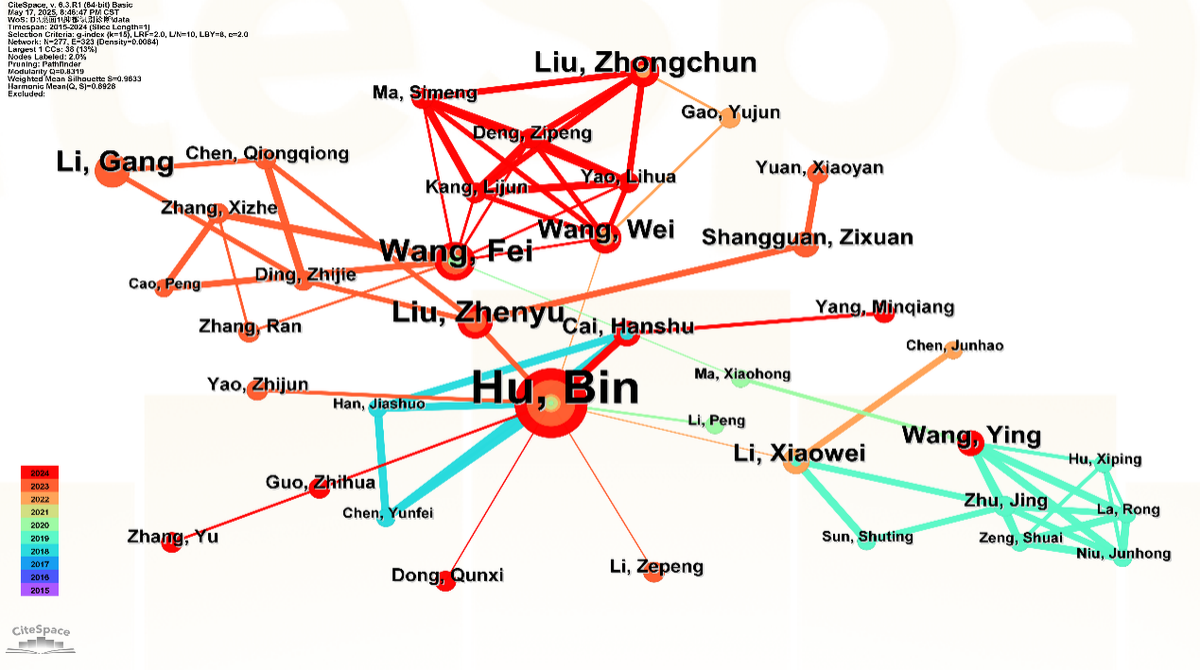
Author coauthorship network in the domain of artificial intelligence–based depression diagnosis from 2015 to 2024.

The author collaboration network within the domain of AI-assisted depression diagnosis from 2015 to 2024, as visualized in Figure 5 and detailed in Table 4, reveals a field marked by productive yet moderately fragmented research clusters. Hu B emerged as the most prolific contributor, with 36 publications since 2018, forming a central node in the network despite a relatively low betweenness centrality (0.02), indicating strong output but limited bridging across research communities. Other active contributors include Acharya UR, Wang F, and Koutsouleris N, with Wang F showing a slightly higher centrality score, reflecting more extensive intergroup collaboration. The network topology features several tightly bound author teams, such as Liu Z–Wang W and Wang F–Yao L, whose intrateam links are dense, but intergroup connectivity remains sparse. Bridge authors such as Cai H and Li X play modest but important roles in linking otherwise disconnected subgroups. The majority of active collaborations have emerged in recent years (2020-2024), reflecting growing interest and integration within the field.

**Table 4:** Author collaboration analysis.

Author	Publication count, n	Betweenness centrality	First year of appearance
Hu, Bin	36	0.02	2018
Acharya, U Rajendra	11	0.00	2015
Koutsouleris, Nikolaos	9	0.00	2018
Wang, Fei	9	0.01	2020
Liu, Zhenyu	8	0.01	2023
Falkai, Peter	7	0.00	2018
Liu, Zhongchun	7	0.00	2022
Li, Gang	7	0.00	2023
Parker, Gordon	6	0.01	2021
Wang, Wei	6	0.01	2022
Wang, Ying	5	0.00	2019
Cao, Bo	5	0.00	2022
Malik, Amir Saeed	5	0.00	2017
Na, Kyoung-Sae	5	0.01	2020
Li, Xiaowei	5	0.00	2019

### Keywords

**Figure 6:**
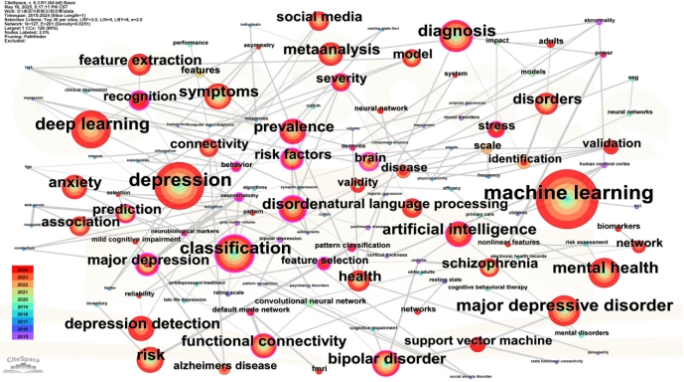
Keyword co-occurrence map.

Figure 6 and Table 5 present the evolving thematic landscape of AI applications in depression diagnosis from 2015 to 2024. The keyword co-occurrence network (Figure 6) prominently displays high-frequency terms such as depression, machine learning, classification, deep learning, and diagnosis, indicating the core research focus areas. The central placement and large node size of depression and machine learning confirm their significant roles as thematic anchors. Additionally, the network highlights close thematic associations among technical methodologies such as neural network, natural language processing, and support vector machine, underscoring a consistent interest in computational techniques for mental health assessments. Simultaneously, terms such as emotion recognition, adolescents, and risk factors indicate an expanding interest in behavioral signal processing, population-specific studies, and clinical contextualization.

**Table 5:** Keyword co-occurrence table.

Keyword	Frequency (approximate)	Notes
depression	High	Core topic
machine learning	High	Key methodology
classification	High	Diagnostic method
deep learning	Moderate	AI subfield
diagnosis	Moderate	Application focus
natural language processing	Moderate	Text analysis method
mental health	Moderate	Context domain
support vector machine	Moderate	Algorithm
risk factors	Moderate	Clinical relevance
emotion recognition	Low-moderate	Behavioral signal processing
adolescents	Low-moderate	Population focus
prevalence	Low	Epidemiology context

**Figure 7:**
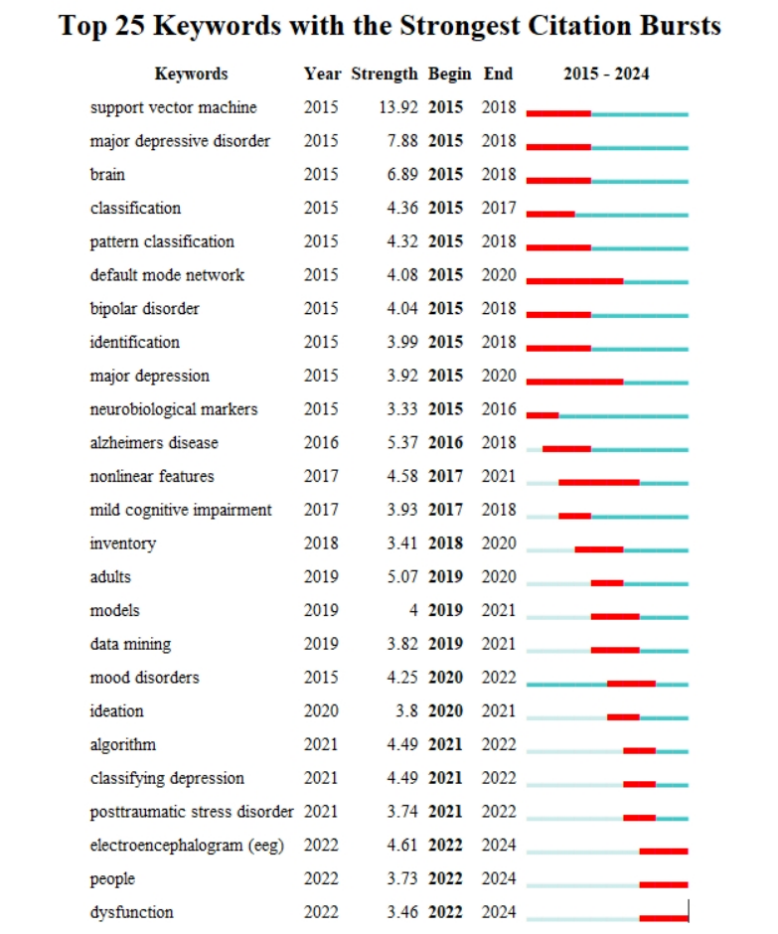
Keyword burst detection.

Figure 7 identifies the top citation burst keywords using Kleinberg’s algorithm, highlighting concepts that received sudden, intense attention over specific periods. The strongest bursts include support vector machine (2015-2018; strength=13.92), major depressive disorder (2015-2018), and brain (2015-2018), signaling foundational methodological and diagnostic focus areas. More recent bursts, such as classifying depression (2021-2022) and electroencephalogram (EEG) (2022-2024), reflect the growing integration of physiological and neural biomarkers into AI models.

**Figure 8:**
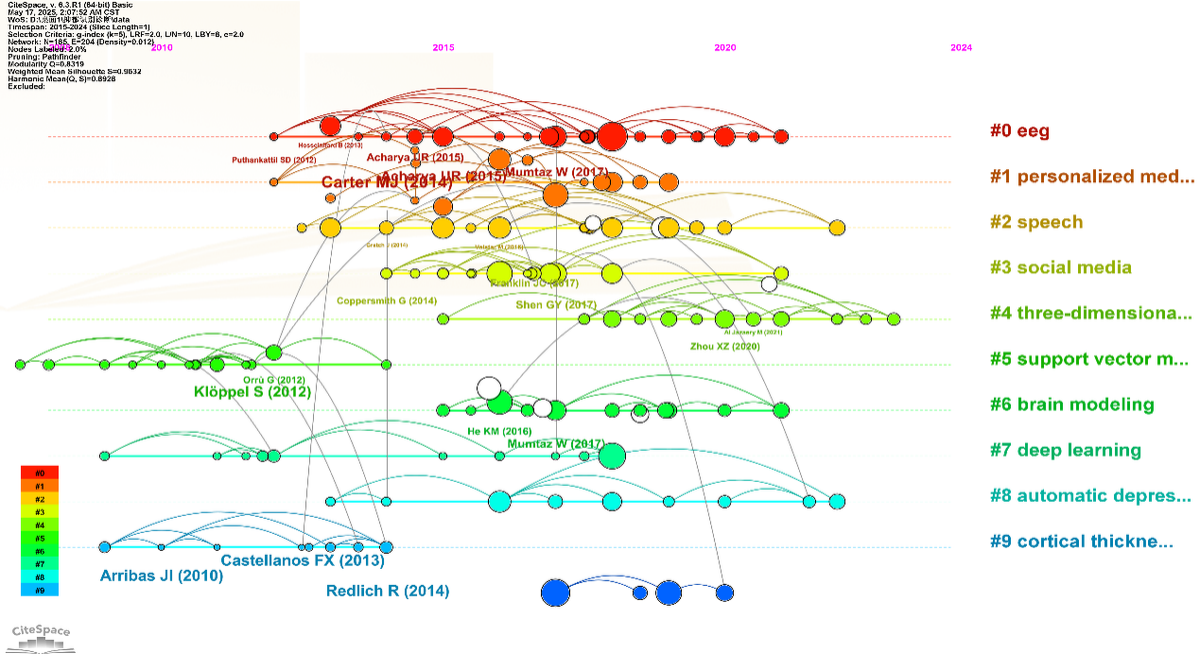
Keyword timeline view.

Figure 8 shows the temporal evolution of keyword clusters from 2015 to 2024. The horizontal axis represents time, and each line traces the development of a thematic cluster. Early-stage clusters like #0 natural language processing and #1 major depressive disorder were dominant between 2015 and 2018, reflecting foundational work. Later clusters, such as #3 machine learning and #4 risk, extended into the 2020s, showcasing an ongoing refinement of predictive modeling techniques and risk assessment tools. More recent clusters like #7 primary care and #9 EEG, emerged around 2022-2024, highlighting the field’s transition toward real-world deployment and biometric signal analysis.

### Reference

**Figure 9:**
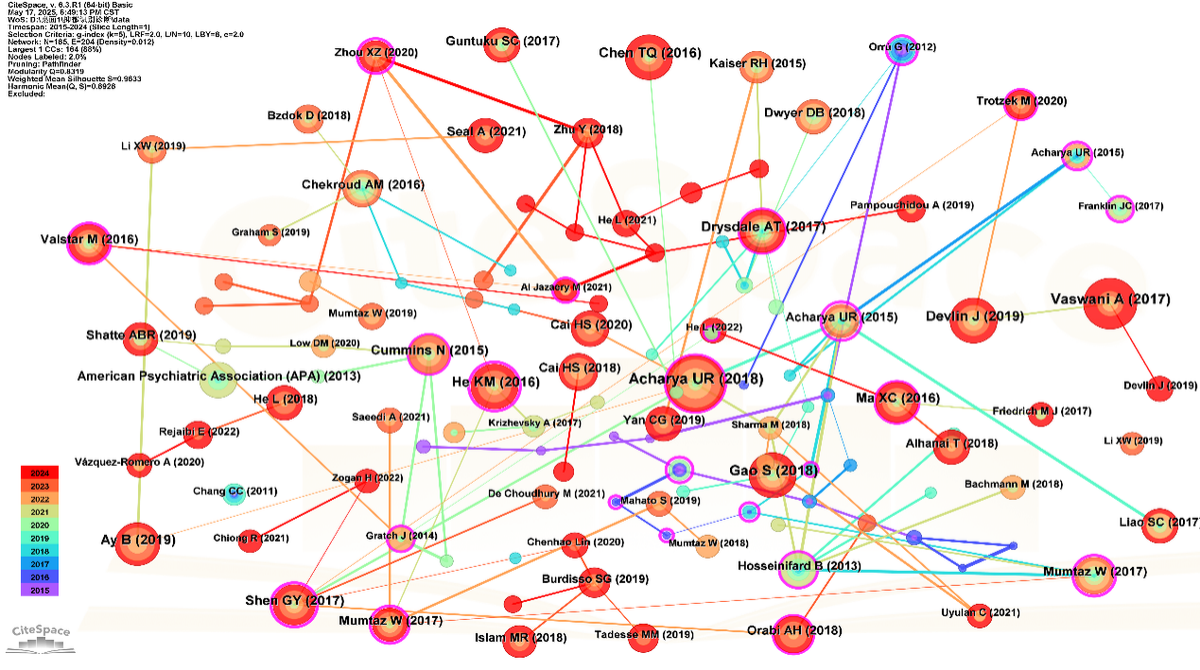
Reference co-citation network.

The reference co-citation network (Figure 9 and Table 6) provides a comprehensive view of the intellectual structure underpinning AI-assisted depression diagnosis research from 2015 to 2024. Several influential studies occupy central positions in the network, notably Acharya UR (2018), Drysdale AT (2017), and Gao S (2018), which serve as key intellectual nodes bridging algorithmic development and clinical application. Acharya UR’s work, with the highest citation count (n=99) and moderate betweenness centrality (0.15), exemplifies this dual impact. Highly central references such as He KM (2016) and Shen GY (2017) act as structural bridges, facilitating interdisciplinary integration across domains including computer vision, psychiatry, and neural networks. The prominence of Vaswani A (2017) and Devlin J (2019) further highlights the increasing adoption of transformer-based natural language processing models, such as BERT for linguistic and semantic analysis in mental health contexts. Temporal color encoding within the network reveals a clear evolution from early emphasis on traditional machine learning approaches, such as support vector machine and EEG-based classification, toward recent advancements in explainable AI, multimodal data fusion, and personalized diagnostics. Overall, the co-citation structure reflects a maturing field grounded in both foundational AI methodologies and clinically oriented research, with key references serving as lasting anchors for emerging scientific paradigms.

**Figure 10:**
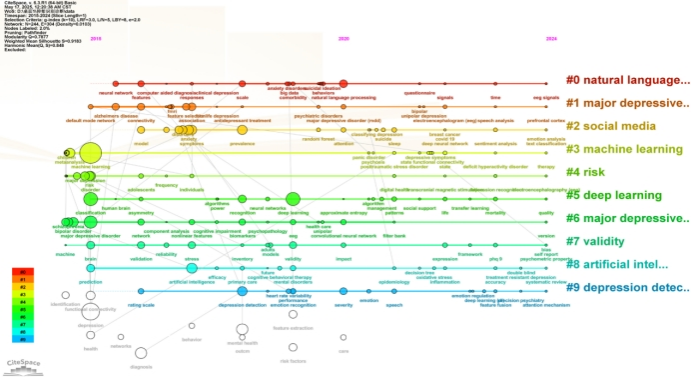
Standardized keyword clusters from co-cited references.

To delineate the developmental trajectory of research on AI-based depression diagnosis, we constructed a reference co-citation timeline using CiteSpace and performed cluster keyword normalization. As illustrated in Figure 10 and Table 7, a total of 10 major clusters (#0 to #9) were identified, each representing a distinct thematic domain within the field.

**Table 6:** Top 10 highly co-cited references in depression and artificial intelligence diagnosis research (2015-2024).

Citation count, n	Betweenness Centrality	Year	Reference
99	0.15	2018	Acharya UR, 2018, COMPUT METH PROG BIO, V161, P103
86	0.02	2017	Vaswani A, 2017, ADV NEUR IN, V30, P0
75	0.03	2018	Gao S, 2018, CNS NEUROSCI THER, V24, P1037
67	0.04	2019	Devlin J, 2019, NAACL HLT, VOL. 1, P4171
67	0.0	2016	Chen TQ, 2016, KDD16, V0, PP785
66	0.19	2017	Drysdale AT, 2017, NAT MED, V23, P28,
65	0.44	2016	He KM, 2016, PROC CVPR IEEE, V0, PP770
61	0.09	2019	Ay B, 2019, J MED SYST, V43, P0
60	0.0	2017	WHO, 2017, DEPRESSION OTHER COM, V0, P0
55	0.49	2017	Shen GY, 2017, IJCAI, V0, P3838

Cluster #0, characterized by keywords such as deep learning, EEG, and feature extraction, reflects the growing integration of neural networks with neurophysiological data for depressive disorder classification. In contrast, clusters #1 and #5 emphasize more conventional machine learning techniques, such as support vector machines and functional magnetic resonance imaging, for biomarker identification and antidepressant response prediction. Emerging clusters such as #2 and #3 highlight the application of multimodal sentiment analysis and social media mining, particularly relevant for early detection in adolescent populations. The prevalence of deep learning-related clusters (#0, #4, #6, and #8) after 2020 signals a paradigm shift from traditional statistical approaches to advanced representation learning methods.

**Table 7:** Cluster evolution analysis.

Cluster ID	Core keywords	Methodological terms	Disease-related terms
#0	deep learning, convolutional neural networks, feature extraction	machine learning, EEG, signal classification, support vector machine	major depressive disorder, depression, beck depression test
#1	machine learning, antidepressant medication, frequency specificity	support vector machine, fMRI, prediction, feature selection	major depressive disorder, mental health
#2	sentiment analysis, speech, multimodal systems	deep learning, facial expression, data analysis, detection	depression, attention deficit hyperactivity disorder
#3	social media, natural language processing, emotion analysis	machine learning, support vector machine, sentiment classification	mental health, depression, psychological disorders
#4	facial expression, deep learning, 3D CNN	feature extraction, spatio-temporal networks, emotion recognition	depression
#5	support vector machine, cortical thickness, surface area	machine learning, multivariate analysis, fMRI	bipolar disorder, post-traumatic stress disorder, pediatric depression
#6	deep learning, task analysis, hidden Markov models	feature extraction, semisupervised learning, convolutional networks	mental disorder
#7	machine learning, cortical thickness, whole-brain connectivity	neuroimaging, longitudinal study, pattern recognition	bipolar disorder, autism spectrum disorder, OCD
#8	deep learning, social networking, sentiment analysis	feature extraction, multimodal fusion, neural networks	depression
#9	pattern recognition, magnetic resonance imaging, surface area	support vector machine, cortical thickness	post-traumatic stress disorder, autism spectrum disorder, bipolar disorder

### Journal

**Figure 11:**
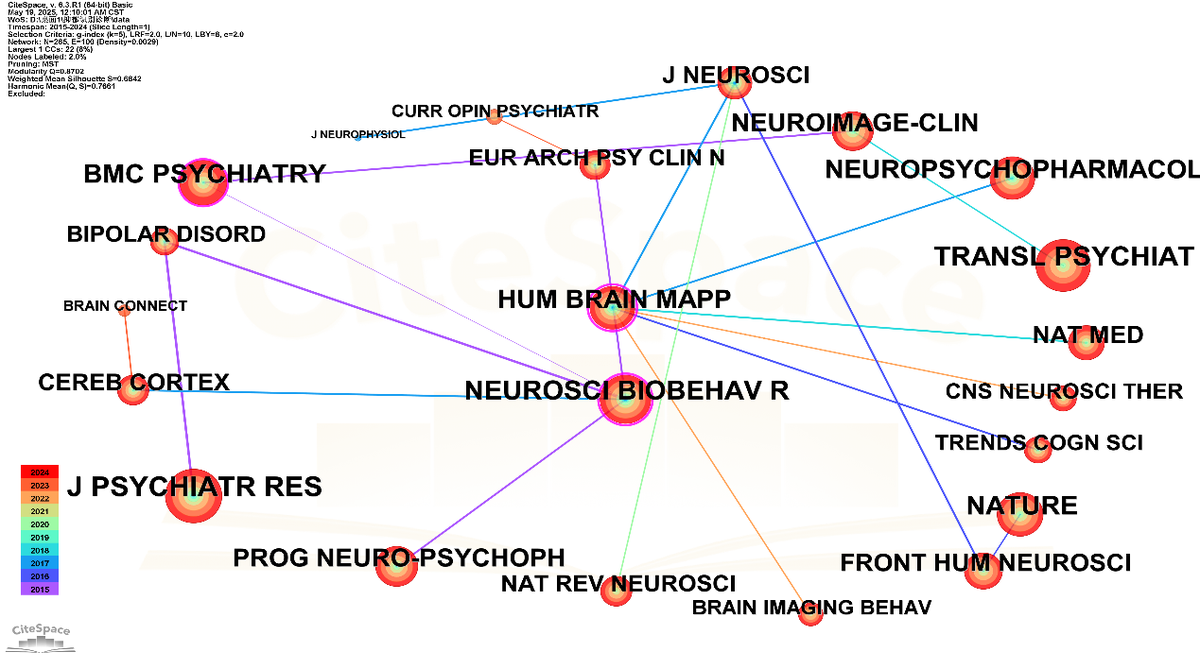
Cited journal co-citation network.

The journal co-citation network (Figure 11 and Table 8) reveals a multidimensional and interdisciplinary intellectual structure within the domain of AI-assisted depression diagnostics from 2015 to 2024. *Journal of Affective Disorders* (J Affect Disord) and *PLOS One* emerged as the most frequently co-cited journals, reflecting their centrality in disseminating both clinical findings and open-access research. In terms of structural influence, *NeuroImage* exhibited the highest betweenness centrality (0.74), underscoring its pivotal role in bridging cognitive neuroscience with AI-based diagnostic modeling. Similarly, *Biological Psychiatry* (Biol Psychiatry) demonstrated strong network centrality (0.55), highlighting its importance in computational psychiatry and methodological innovation. The presence of high-impact general science journals, such as *Nature* and *Trends in Cognitive Sciences*, along with translational platforms such as *Translational Psychiatry* (Transl Psychiatry) and *Progress in Neuro-Psychopharmacology & Biological Psychiatry*, indicates a dynamic flow of ideas across disciplinary boundaries. Moreover, engineering-oriented outlets such as IEEE Access signal the increasing role of computational modeling and machine learning frameworks in mental health research. The overall structure of the network suggests a research landscape shaped by both foundational psychiatric discourse and emerging technological approaches.

**Table 8:** High-impact journal co-citation network in depression research.

Cited Journal	Count	Centrality	Year	Remark
J Affect Disord^a^	1008	0.07	2015	Clinical core journal
PLOS One	973	0.03	2015	Cross-disciplinary open access
Schizophr Res^b^	637	0.00	2017	Data corrected
Lancet	572	0.00	2015	Global epidemiology
Am J Psychiatry^c^	559	0.02	2015	DSM-5^d^ alignment
Biol Psychiatry^e^	549	0.55	2015	Computational psychiatry
NeuroImage	435	0.74	2015	Neuroimaging–artificial intelligence fusion
IEEE Access	451	—^f^	2019	Engineering methods
Transl Psychiatry^g^	407	0.01	2018	Emerging focus

^a^J Affect Disord: Journal of Affective Disorders.

^b^Schizophr Res: *Schizophrenia Research*.

^c^Am J Psychiatry: American Journal of Psychiatry.

^d^DSM-5: Diagnostic and Statistical Manual of Mental Disorders (Fifth Edition).

^e^Biol Psychiatry: *Biological Psychiatry*.

^f^Not available.

^g^Transl Psychiatry: *Translational Psychiatry*.

## Discussion

### Summary of Key Findings

Previous bibliometric studies in mental health informatics have primarily focused on broader digital psychiatry applications or specific technological subdomains, leaving a comprehensive analysis of AI-driven depression diagnosis unexplored. Ramos-Lima et al [16] examined machine learning applications in psychiatry broadly but did not provide a focused analysis of depression-specific research. Similarly, Abd-Alrazaq et al [17] analyzed chatbot applications in mental health, representing only a narrow subset of AI technologies. This focused comparison confirms that this study is not only filling a knowledge gap but also providing a more nuanced understanding of the unique developmental trajectory of AI-driven depression research.

This study presents a comprehensive bibliometric mapping of AI research in depression diagnosis over the past decade, revealing several key structural and thematic trends. Keyword network analysis demonstrates the persistent influence of core computational methods, such as machine learning, deep learning, and support vector machines, while emerging terms such as multimodal fusion, facial expression recognition, and EEG-based detection signal a clear shift toward integrative, noninvasive diagnostic approaches. Coauthorship and institutional analyses reflect a moderately collaborative but thematically focused research community, with central contributions from scholars such as Zhang Y, Wang Y, and Li H, and leading institutions including the University of Toronto, the Chinese Academy of Sciences, and Harvard Medical School. At the national level, China and the United States dominate in volume, yet rising centrality from countries like India, Saudi Arabia, and Pakistan suggests increasing geographic diversification. Co-citation networks of references and journals further delineate the intellectual foundations of the field, anchored in neuroimaging, affective computing, and machine learning–based psychiatric evaluation. These patterns collectively depict a field undergoing rapid technological advancement, with expanding interdisciplinary integration and growing global participation.

### In-Depth Interpretation of Core Findings

#### Temporal Growth and Thematic Expansion

The exponential surge in scholarly output and citation impact from 2015 to 2024 (Figure 1 and Table 1) stands as a testament to the escalating scientific interest and investment in AI applications for depression. This trajectory is not linear; rather, it reflects a period of accelerated innovation and widespread adoption of computational methods to address a persistent global health challenge. Several interconnected factors elucidate this rapid expansion. The maturation of foundational AI algorithms, particularly advancements in deep learning, machine learning, and natural language processing, has provided researchers with increasingly powerful and accessible tools for complex data analysis [18]. The advent of graphics processing units has fundamentally transformed computational research by enabling the processing of large datasets and complex deep learning models [19]. Graphic processing units, originally designed for graphics applications, have become crucial for accelerating deep learning development because of their highly parallel processing capabilities [20]. This technological advancement, combined with the availability of significantly larger datasets from big data growth, has enabled breakthroughs across machine learning, AI, and computer vision that were previously constrained by computational limitations [21]. Furthermore, the growing availability and proliferation of diverse multimodal data sources, ranging from neuroimaging and electrophysiological signals to speech patterns, facial expressions, and digital footprints, have supplied the essential fuel for AI model development [22]. This expansion of data sources has enabled researchers to construct more nuanced diagnostic frameworks, fostering a critical transition from traditional assessment tools to dynamic, data-driven paradigms [23]. Crucially, the persistent limitations of conventional subjective diagnostic methods for depression, coupled with the immense global burden of the disorder, have created a critical demand for objective, scalable, and early detection solutions [24]. The scientific community’s response to this unmet clinical need, empowered by technological advances, has catalyzed this remarkable growth. This accelerated trajectory mirrors similar patterns observed in other rapidly emerging, technology-driven subfields within digital health, where initial foundational work gives way to explosive growth upon critical technological breakthroughs and demonstrable early successes.

#### Methodological Evolution and Thematic Reorientation

The analysis of the keyword network provides a compelling narrative of the field’s methodological evolution. The sustained dominance of high-frequency terms such as machine learning, deep learning, and support vector machine underscores the foundational and enduring reliance on classical and contemporary computational paradigms for developing diagnostic models. Early-stage studies predominantly used classical machine learning techniques such as support vector machines and random forests, often relying on structured clinical features [25,26]. However, the more recent burst detection analysis offers critical foresight into emerging frontiers. The prominent bursts of terms like multimodal fusion, facial expression recognition, and EEG-based detection post-2020 unequivocally signal a significant methodological pivot. This trend highlights a progressive and strategic shift away from reliance on single data modalities toward integrative, multimodal approaches [27]. This shift is not an isolated phenomenon but is deeply interconnected with the overall technological breakthroughs in AI. The widespread availability of high-performance computing resources, the accessibility of large-scale datasets, and the maturity of deep learning frameworks have collectively provided the technical foundation for more sophisticated models to handle high-dimensional, multimodal data [28]. For instance, deep learning models based on unstructured data such as speech, images, and text can automatically extract complex features, demonstrating the potential to surpass traditional methods in objective diagnosis [29,30]. Such fusion techniques aim to capture a richer, more comprehensive representation of depressive symptoms by combining diverse data streams, thereby potentially overcoming the limitations of individual data types [31]. Furthermore, the emphasis on facial expression recognition [32] and EEG-based detection [33] indicates a strong drive toward developing noninvasive and objective diagnostic modalities, which hold immense promise for enhancing the scalability and reducing the burden of depression screening in clinical and real-world settings. This evolution reflects the field’s continuous pursuit of more robust, accurate, and practical AI-driven diagnostic solutions, reorienting toward capturing the heterogeneity of depression through diverse data inputs.

#### Collaboration Dynamics: Authors, Institutions, and Countries

The intricate patterns within the author-, institutional-, and country-level collaboration networks collectively shed light on the structural characteristics of the research community. While the author coauthorship network demonstrated a fragmented yet thematically coherent research community, it suggests that researchers are focused on similar problems but perhaps not always in highly interconnected teams. The emergence of a limited number of prolific authors (eg, Zhang Y, Wang Y, and Li H) as significant contributors is typical for rapidly advancing fields, in which a few leading experts often shape the initial directions and intellectual output. However, the moderate overall collaboration density and the implications of betweenness centrality measures—indicating a need for broader cross-group integration—underscore a potential bottleneck in knowledge diffusion and the synergistic generation of novel ideas. Enhancing intergroup collaboration could accelerate breakthroughs and foster a more robust research ecosystem.

At the institutional level, the prominence of entities such as the University of Toronto, Chinese Academy of Sciences, and Harvard Medical School, marked by their high publication volume and strategic centrality, reaffirms their role as global leaders in scientific innovation. These institutions likely possess the requisite expertise, resources, and infrastructure to drive high-impact research in this complex interdisciplinary domain. Nevertheless, the observable trend of institutional cooperation primarily confined within national boundaries signifies a notable limitation in the current collaborative landscape. This insular tendency, while potentially efficient for domestic resource pooling, may impede the optimal exchange of diverse perspectives, methodologies, and data from different health care systems and populations, thus limiting the global generalizability and impact of findings.

Correspondingly, the country-level analysis further solidifies this observation. The dominance of China and the United States, collectively contributing over 60% of the total output, reflects their significant investments in AI research and mental health initiatives. This concentration underscores their leading roles in shaping the trajectory of AI for depression diagnosis. Intriguingly, the increasing centrality of emerging economies such as India, Pakistan, and Saudi Arabia in recent years signals a nascent but important geographic decentralization of research leadership. This shift suggests a growing global interest and capacity in leveraging AI for mental health, particularly in regions that may face unique challenges in depression diagnosis and treatment. Furthermore, the roles of Canada and Singapore as pivotal bridges in the international collaboration network, indicated by their high betweenness centrality, highlights their strategic importance in fostering cross-national partnerships. These nations thus serve as strategic connectors that facilitate transregional information flow and collaboration.

#### Intellectual Foundations and Disciplinary Integration

The co-citation analysis of journals and cited references provides a robust mapping of the field’s intellectual bedrock and its disciplinary integration. The consistent appearance of J Affect Disord, Biol Psychiatry, NeuroImage, and Transl Psychiatry as foundational contributors strongly indicates that this domain draws heavily from established clinical psychiatry and neuroscience. These journals span psychiatry, neuroscience, neuroimaging, and computational sciences, underscoring the inherently interdisciplinary nature of AI-based depression research. The high centrality of NeuroImage, in particular, signifies its crucial bridging role between advanced neuroimaging techniques and AI-driven diagnostic methodologies. Similarly, Biological Psychiatry’s strong centrality underscores the continuing influence of biological psychiatry, anchoring AI research in a robust understanding of underlying pathophysiological mechanisms.

The gaining momentum of IEEE Access and Translational Psychiatry further reflects a significant methodological and translational shift. IEEE Access, representing engineering and computer science, indicates a burgeoning integration of computational rigor into psychiatric research, signaling a strong move from conceptual experimentation toward scalable, clinically viable solutions. *Translational Psychiatry*, conversely, highlights a strong emphasis on moving basic AI research findings closer to clinical application and real-world impact. While traditional psychiatric journals such as *American Journal of Psychiatry* and *Schizophrenia Research* remain foundational, their relatively lower structural influence in the co-citation network (compared with their direct citation counts, which may still be high) suggests that the most dynamic, cutting-edge intellectual development, particularly regarding novel AI methodologies, now often occurs at the intersection of more diverse, technologically advanced, and translational journals. The observed strong modularity (Q>0.7) and high silhouette scores (S>0.8) of the identified knowledge clusters further reinforce that, despite its interdisciplinary nature, the field comprises well-defined, semantically cohesive knowledge domains, ensuring specialized development within broader integration.

### Theoretical and Practical Implications

#### Theoretical Implications

This study presents a series of important theoretical contributions to the intersection of AI and depression diagnosis. First, by conducting a comprehensive bibliometric analysis, we offer a nuanced and systematic understanding of the intellectual trajectory in AI-driven depression research over the past decade. By mapping key research themes, authors, and institutions, this work sheds light on the interdisciplinary nature of the field, with significant shifts observed in both methods and focus areas. Early studies primarily used traditional machine learning algorithms, such as support vector machines and decision trees, focusing mainly on structured clinical data [34]. However, recent research has seen a clear pivot toward more sophisticated models, including deep learning approaches and multimodal integration [35]. These developments reflect a growing understanding of depression as a multifactorial disorder that requires diagnostic frameworks capable of addressing the diversity and complexity of depressive symptoms [36]. This theoretical progression opens new avenues for future research that integrates machine learning with neuroimaging, social behavior, and longitudinal assessments, thereby enriching our conceptualization of depression’s pathophysiology and diagnostic parameters.

Second, the identification of emerging keywords and research bursts, particularly those centered on multimodal systems, affective computing, and real-time data analysis, suggests a shift toward a more dynamic and contextually relevant approach to depression diagnosis. These findings encourage the development of new theoretical models that transcend laboratory-based research and seek to incorporate ecological data from naturalistic settings. Future theories should explore how real-time AI systems, powered by diverse data streams, can accurately reflect the complexity of depressive behaviors across different populations, environments, and life stages [37].

#### Bridging the Gap Between Research and Clinical Impact

In practical terms, the findings from this study have profound implications for both clinical practice and the further development of AI tools for depression diagnosis, while also illuminating a critical disconnect between publication patterns and the actual clinical impact of these tools [38]. The increasing use of deep learning and multimodal systems, while promising, remains largely confined to academic settings. This disparity underscores several significant translational challenges that the field must address to ensure that academic advancements lead to tangible progress in mental health care [39]. The increasing application of deep learning and multimodal systems offers substantial promise for improving diagnostic accuracy, personalization, and scalability [40]. With the integration of data sources such as facial expressions, speech patterns, and biometric signals, AI-driven diagnostic tools could enable clinicians to obtain real-time, objective assessments that are less dependent on patient self-reporting and subjective judgment. Such tools can potentially streamline diagnostic processes in clinical settings, especially where resources are limited and mental health professionals are in short supply [41]. Furthermore, AI models could provide an efficient way of monitoring treatment progress, enabling timely interventions in cases where patients may not fully disclose their symptoms [42]. However, a key obstacle is the lack of robust, large-scale clinical trials and regulatory approval, which are essential for clinical validation [43].

The analysis of global collaboration networks also has significant practical implications. While countries such China and the United States have dominated publication output in this field, emerging economies such as India, Saudi Arabia, and Pakistan are becoming increasingly influential in global AI depression research. This trend suggests a decentralization of research power and highlights the importance of fostering international collaboration, especially in low-resource settings. By promoting cross-border partnerships, researchers and policy makers can help bridge existing gaps in mental health care access, ensuring that AI technologies are used in diverse contexts and are adapted to local challenges.

In essence, for the academic trends identified in this study to translate into meaningful clinical implications, future research must pivot from solely focusing on algorithmic performance to addressing these critical translational obstacles. It is crucial that ethical issues such as data privacy, transparency, and algorithmic fairness are addressed [44]. As AI technologies evolve, they must be designed to adhere to ethical guidelines that protect patient data and ensure the fair and unbiased application of machine learning models [45,46]. Future research should prioritize the development of frameworks that address these ethical concerns, as they are critical for the broader adoption and acceptance of AI in mental health care.

### Limitations

This study has several limitations. First, the analysis was restricted to publications indexed in the WoSCC and written in English, which may have excluded relevant studies published in other databases or languages. While this restriction was necessary to ensure data consistency and compatibility with bibliometric tools, future research could incorporate additional sources such as PubMed, Scopus, or IEEE Xplore to enhance comprehensiveness. Second, although our search strategy used a rigorously constructed topic string encompassing the most commonly used clinical and technical terms (eg, depression, major depressive disorder, and MDD), it did not extend to broader constructs such as mood disorders. This narrower focus was intentional to ensure conceptual clarity and avoid semantic ambiguity; however, it may have resulted in the exclusion of some relevant literature. Finally, the bibliometric approach inherently provides a descriptive overview rather than a mechanistic explanation of research dynamics; thus, the findings should be interpreted as a mapping of publication patterns rather than definitive evidence of clinical impact.

### Future Directions and Outlook

Building upon the intricate patterns identified in this bibliometric analysis and their significant implications, the trajectory of AI applications in depression detection and diagnosis points toward several promising avenues for future research and development. Addressing these crucial directions will be instrumental in translating technological advancements into impactful, patient-centered clinical solutions.

One primary direction lies in the enhancement of multimodal data fusion techniques and the concurrent development of explainable AI models. While the field has seen a significant shift toward integrating diverse data streams—ranging from neuroimaging and physiological signals to behavioral patterns and linguistic markers from social media—future efforts should focus on more sophisticated fusion algorithms. These advanced algorithms must not only combine disparate modalities effectively but also determine the relative contribution of each data type to diagnostic accuracy.

A second critical frontier involves the longitudinal validation and rigorous personalization of AI models within diverse real-world clinical contexts. Moving beyond current cross-sectional studies and controlled laboratory environments, future research should prioritize large-scale, prospective longitudinal investigations. These studies would track individuals over extended periods using AI-driven monitoring tools, facilitating the development of more precise predictive models for depression onset, relapse, and individual treatment response, thereby advancing toward a truly precision psychiatry approach. Furthermore, the generalizability and scalability of AI diagnostic tools must be rigorously tested across diverse demographics, cultural backgrounds, and varied health care settings, including those in resource-constrained regions. This necessitates the creation of more ethnically, socioeconomically, and clinically diverse datasets, potentially leveraging enhanced global collaborations to ensure equitable and effective deployment of AI solutions worldwide.

Finally, the burgeoning field must proactively address the complex interdisciplinary and ethical challenges inherent in this rapidly evolving domain. Future research initiatives should emphasize fostering deeper synergistic collaborations among a broad spectrum of experts, including AI specialists, psychiatrists, neuroscientists, computational linguists, social scientists, and ethicists, to collectively co-design and co-validate responsible AI solutions. The ultimate outlook for AI in depression diagnosis lies in its responsible, ethical, and seamless integration into a human-centered health care system, transforming it from a mere diagnostic aid into a truly preventive, personalized, and universally accessible mental health companion.

### Conclusions

This comprehensive bibliometric and visual analysis systematically mapped the evolving landscape of AI applications in depression detection and diagnosis from 2015 to 2024. Our investigation revealed an exponential growth trajectory and a significant methodological evolution toward multimodal data fusion and objective diagnostic markers, highlighting a dynamic global research collaboration network and the field’s inherent multidisciplinarity rooted in computational psychiatry, neuroscience, and engineering. In essence, this study provides a robust quantitative overview of the field’s thematic expansion and collaborative structures, offering crucial guidance for future research that emphasizes enhanced interdisciplinary collaboration, the development of explainable and ethically robust AI models, and their rigorous validation for equitable clinical translation. These findings hold considerable promise for advancing global mental health diagnostics and may also inform evidence-based policymaking in the development, evaluation, and implementation of AI-assisted diagnostic tools in mental health services.

## Abbreviations

AI: artificial intelligence
Biol Psychiatry: Biological Psychiatry
EEG: electroencephalogram
F: frequency
J Affect Disord: Journal of Affective Disorders
Q: modularity
S: silhouette score
Transl Psychiatry: Translational Psychiatry
WHO: World Health Organization

## Data Availability

The datasets generated or analyzed during this study are available from the corresponding author on reasonable request.

## References

[1] Forbes A, Keleher MR, Venditto M, DiBiasi F (2023). Assessing patient adherence to and engagement with digital interventions for depression in clinical trials: systematic literature review. J Med Internet Res.

[2] GBD 2017 Disease and Injury Incidence and Prevalence Collaborators (2018). Global, regional, and national incidence, prevalence, and years lived with disability for 354 diseases and injuries for 195 countries and territories, 1990-2017: a systematic analysis for the Global Burden of Disease Study 2017. Lancet.

[3] Friedrich MJ (2017). Depression is the leading cause of disability around the world. JAMA.

[4] Caan W (2015). The global crisis of depression: the low of the 21st century?. Perspect Public Health.

[5] Zimmerman M, Martinez JH, Young D, Chelminski I, Dalrymple K (2013). Severity classification on the hamilton depression rating scale. J Affect Disord.

[6] Furukawa TA (2010). Assessment of mood: guides for clinicians. J Psychosom Res.

[7] Bilello JA (2016). Seeking an objective diagnosis of depression. Biomark Med.

[8] Squires M, Tao X, Elangovan S, Gururajan R, Zhou X, Acharya UR, Li Y (2023). Deep learning and machine learning in psychiatry: a survey of current progress in depression detection, diagnosis and treatment. Brain Inform.

[9] Lynch CJ, Gunning FM, Liston C (2020). Causes and consequences of diagnostic heterogeneity in depression: paths to discovering novel biological depression subtypes. Biol Psychiatry.

[10] Ahmed I, Brahmacharimayum A, Ali RH, Khan TA, Ahmad MO (2025). Explainable AI for depression detection and severity classification from activity data: development and evaluation study of an interpretable framework. JMIR Ment Health.

[11] Baydili I, Tasci B, Tasci G (2025). Artificial intelligence in psychiatry: a review of biological and behavioral data analyses. Diagnostics (Basel).

[12] Teferra BG, Rueda A, Pang H, Valenzano R, Samavi R, Krishnan S, Bhat V (2024). Screening for depression using natural language processing: literature review. Interact J Med Res.

[13] Jin N, Ye R, Li P (2025). Diagnosis of depression based on facial multimodal data. Front Psychiatry.

[14] Chen C, Hu Z, Liu S, Tseng H (2012). Emerging trends in regenerative medicine: a scientometric analysis in CiteSpace. Expert Opin Biol Ther.

[15] Chen C (2004). Searching for intellectual turning points: progressive knowledge domain visualization. Proc Natl Acad Sci U S A.

[16] Ramos-Lima LF, Waikamp V, Antonelli-Salgado T, Passos IC, Freitas LHM (2020). The use of machine learning techniques in trauma-related disorders: a systematic review. J Psychiatr Res.

[17] Abd-Alrazaq AA, Rababeh A, Alajlani M, Bewick BM, Househ M (2020). Effectiveness and safety of using chatbots to improve mental health: systematic review and meta-analysis. J Med Internet Res.

[18] Mottaghi-Dastjerdi N, Soltany-Rezaee-Rad M (2024). Advancements and applications of artificial intelligence in pharmaceutical sciences: a comprehensive review. Iran J Pharm Res.

[19] Zhang Z, Zhu J, Guo Z, Zhang Y, Li Z, Hu B (2024). Natural language processing for depression prediction on sina weibo: method study and analysis. JMIR Ment Health.

[20] LeCun Y, Bengio Y, Hinton G (2015). Deep learning. Nature.

[21] Saleh S (2020). Embed Selforganising Syst.

[22] Ramanarayanan V (2024). Multimodal technologies for remote assessment of neurological and mental health. J Speech Lang Hear Res.

[23] Ren G, Krawetz R (2015). Applying computation biology and "big data" to develop multiplex diagnostics for complex chronic diseases such as osteoarthritis. Biomarkers.

[24] Bilello JA (2016). Seeking an objective diagnosis of depression. Biomark Med.

[25] Yu W, Sun T, Hsu K, Wang C, Chien S, Tsai C, Yang Y (2024). Comparative analysis of machine learning algorithms for Alzheimer's disease classification using EEG signals and genetic information. Comput Biol Med.

[26] Guetari R, Ayari H, Sakly H (2023). Computer-aided diagnosis systems: a comparative study of classical machine learning versus deep learning-based approaches. Knowl Inf Syst.

[27] Steyaert S, Pizurica M, Nagaraj D, Khandelwal P, Hernandez-Boussard T, Gentles AJ, Gevaert O (2023). Multimodal data fusion for cancer biomarker discovery with deep learning. Nat Mach Intell.

[28] Lac L, Leung CK, Hu P (2024). Computational frameworks integrating deep learning and statistical models in mining multimodal omics data. J Biomed Inform.

[29] Dattola S, Ielo A, Varone G, Cacciola A, Quartarone A, Bonanno L (2025). Frontotemporal dementia: a systematic review of artificial intelligence approaches in differential diagnosis. Front Aging Neurosci.

[30] Cüce F, Tulum G, Isik MI, Jalili M, Girgin G, Karadaş Ö, Baş N, Özcan B, Savaşci Ü, Şakir S, Karadaş AÖ, Teomete E, Osman O, Rasheed J (2025). A novel MRI-based deep learning-radiomics framework for evaluating cerebrospinal fluid signal in central nervous system infection. Front Med (Lausanne).

[31] Sui J, Zhi D, Calhoun VD (2023). Data-driven multimodal fusion: approaches and applications in psychiatric research. Psychoradiology.

[32] Porter-Vignola E, Booij L, Bossé-Chartier G, Garel P, Herba CM (2021). Emotional facial expression recognition and depression in adolescent girls: associations with clinical features. Psychiatry Res.

[33] Habib A, Vaniya SN, Khandoker A, Karmakar C (2024). MDDBranchNet: a deep learning model for detecting major depressive disorder using ECG signal. IEEE J Biomed Health Inform.

[34] Lee K, Ham B (2022). Machine learning on early diagnosis of depression. Psychiatry Investig.

[35] Yang S, Cui L, Wang L, Wang T, You J (2024). Enhancing multimodal depression diagnosis through representation learning and knowledge transfer. Heliyon.

[36] Kennedy SH, Ceniti AK (2018). Unpacking major depressive disorder: from classification to treatment selection. Can J Psychiatry.

[37] Levkovich I (2025). Is artificial intelligence the next co-pilot for primary care in diagnosing and recommending treatments for depression?. Med Sci (Basel).

[38] Reyna MA, Nsoesie EO, Clifford GD (2022). Rethinking algorithm performance metrics for artificial intelligence in diagnostic medicine. JAMA.

[39] Vaidyam A, Halamka J, Torous J (2022). Enabling research and clinical use of patient-generated health data (the mindLAMP Platform): digital phenotyping study. JMIR Mhealth Uhealth.

[40] Shiyam Sundar LK, Muzik O, Buvat I, Bidaut L, Beyer T (2021). Potentials and caveats of AI in hybrid imaging. Methods.

[41] Ruzek JI, Yeager CM (2017). Internet and mobile technologies: addressing the mental health of trauma survivors in less resourced communities. Glob Ment Health (Camb).

[42] Mendes Serrão E, Klug M, Moloney BM, Jhaveri A, Lo Gullo R, Pinker K, Luker G, Haider MA, Shinagare AB, Liu X (2023). Current status of cancer genomics and imaging phenotypes: what radiologists need to know. Radiol Imaging Cancer.

[43] Koutsouleris N, Hauser TU, Skvortsova V, De Choudhury M (2022). From promise to practice: towards the realisation of AI-informed mental health care. Lancet Digit Health.

[44] Weiner EB, Dankwa-Mullan I, Nelson WA, Hassanpour S (2025). Ethical challenges and evolving strategies in the integration of artificial intelligence into clinical practice. PLOS Digit Health.

[45] Singhal M, Gupta L, Hirani K (2023). A comprehensive analysis and review of artificial intelligence in Anaesthesia. Cureus.

[46] Mennella C, Maniscalco U, De Pietro G, Esposito M (2024). Ethical and regulatory challenges of AI technologies in healthcare: a narrative review. Heliyon.

